# Visible light-driven photocatalysts, quantum chemical calculations, ADMET-SAR parameters, and DNA binding studies of nickel complex of sulfadiazine

**DOI:** 10.1038/s41598-023-42668-z

**Published:** 2023-09-15

**Authors:** Sachin B. Pandya, Bhavesh N. Socha, Rahul P. Dubey, Urmila H. Patel, R. H. Patel, Bhupesh S. Bhatt, Parth Thakor, Sanjay Bhakhar, Nikhil Vekariya, Jignesh Valand

**Affiliations:** 1 Department of Physics, Sardar Patel University, Vallabh Vidyanagar, Anand, 388120 Gujarat India; 2 Department of Chemistry, Sardar Patel University, Vallabh Vidyanagar, Anand, 388120 Gujarat India; 3 Department of Materials Science, Sardar Patel University, Vallabh Vidyanagar, Anand, 388120 Gujarat India; 4 Vivekanand P.G. College, Govind Guru Tribal University, Banswara, Rajasthan India; 5 Bapubhai Desaibhai Patel Institute of Paramedical Sciences, Charotar University of Science and Technology, Changa, India

**Keywords:** Drug discovery, Chemistry

## Abstract

A 3D-supramolecular nickel integrated Ni-SDZ complex was synthesized using sodium salt of sulfadiazine as the ligand and nickel(II) acetate as the metal salt using a condensation process and slow evaporation approach to growing the single crystal. The metal complex was characterized for its composition, functional groups, surface morphology as well as complex 3D structure, by resorting to various analytical techniques. The interacting surface and stability as well as reactivity of the complex were carried out using the DFT platform. From ADMET parameters, human Intestinal Absorbance data revealed that the compound has the potential to be well absorbed, and also Ni-SDZ complex cannot cross the blood–brain barrier (BBB). Additionally, the complex's DNA binding affinity and in-vivo and in-vitro cytotoxic studies were explored utilizing UV–Vis absorbance titration, viscosity measurements, and *S. pombe* cells and brine shrimp lethality tests. In visible light radiation, the Ni-SDZ complex displayed exceptional photo-degradation characteristics of approximately 70.19% within 70 min against methylene blue (MB).

## Introduction

Organometallic compounds have piqued the interest of researchers due to their extraordinary properties such as large surface area, crystallinity, and antibacterial activity^1–3^. Sulfonamides, an organic sulfur-containing compound radical –SO_2_NH_2_^–^, derived from chiefly sulfanilamide, having a similar molecular structure to P-aminobenzoic acid (PABA), are capable of interfering with the metabolic processes in bacteria. They act as antimicrobial agents by inhibiting bacterial growth and activity and are called sulfa drugs^4^. Sulfonamides are used in the prevention and treatment of bacterial infections, diabetes mellitus, edema, hypertension, gout, allergies, and cough, as well as antifungal and antimalarial^5–16^ Sulfadiazine**,** a member of the triple sulfa drug family (sulfamerazine, sulfamethazine, and sulfadiazine) used as an antibacterial agent. In combination, sulfadiazine and pyrimethamine are used to treat toxoplasmosis, a disease caused by *Toxoplasma gondii*^17,18^ It is capable of inhibiting the growth or reproduction of bacteria but not killing the bacteria. It blocks the synthesis of bacterial dihydrofolic acid and therefore Figure S1 (Supplementary information). The lack of access to safe drinking water is one of the major health concerns of modern life. One of the most common compounds that renders water unsafe for drinking is industrial dyes. Methylene blue (MB), one of these dyes, poses the greatest risk to human health and environmental security since it is poisonous, carcinogenic, and non-biodegradable. It is typically discharged into natural water sources, endangering the health of both people and other living things. Therefore, it is necessary to create an effective, ecologically acceptable method of eliminating MB and other dyes from wastewater. A popular advanced oxidation method for removing dye is photodegradation. The full mineralization of the dye into simple, non-toxic species has the advantage of potentially lowering the cost of processing. Transition metal complexes have a wide range of applications in materials and biological sciences^19–22^. In the search for novel therapy against resistant organisms, the existing drug attempted to modify by incorporating metal on it. The presence of metal ions helps to accelerate drug action^23–28^. The metal complexes of sulfadiazine, especially silver sulfadiazine used in burn therapy, and zinc sulfadiazine used in the prevention of bacterial infection in burned animals^29–32^. The intercalation of transition metal complexes of sulfonamide derivatives into DNA has been a topic of major bioinorganic interest in the past two decades and continues to obtain much attentions^33–38^. Also, the transition metal complex and its nanocomposite-based photocatalysts have recently been established for the breakdown of the dye contained in wastewater. Because of energy and environmental concerns, the usefulness of metal complexes as a catalyst for photochemical processes has been widely researched. Metal complexes and their nanocomposite-based photocatalysts include UV-active and visible-responsive photocatalysts that are used to degrade organic contaminants. Transition metal-based complexes and composites are explored in visible and UV-active photocatalysis. This study aimed to assess the effectiveness of the sprd-free ligand once the metal is incorporated into it, to enhance both the biological activity and the photodegradation potential for a chosen organic dye, methylene blue (MB), within an aqueous solution. The metal complex (Ni-SDZ) properties such as Homo–Lumo energy have been calculated to get a better understanding of the properties of the Ni-SDZ molecule. Also, NBO analysis is the study of an intramolecular delocalization of hyper-conjugation. Hirshfeld surface analysis and their related 2D fingerprint plots evaluated the intermolecular interactions. Furthermore, we conclude from the magnetic behaviour of the nickel complex of sulfadiazine, the coordination environment surrounding nickel is distorted octahedral. The interaction of the nickel complex of sulfadiazine with DNA has improved much attention due to their possible applications as new therapeutic agents. In the present work, we have concluded the nature of ligands is of dominant importance in the interaction of the complex with DNA molecule. The DNA-binding of the complex has been studied using absorption titration and viscosity measurements are also reported.

## Experimental details

### Materials and instrumentations

Sodium salt of sulfadiazine (SDZ) (Sigma, purity of the compound was > 99%), nickel(II) acetate (Alfa Aezar, purity of the compound was > 99%), and other reagents are of the highest grade commercially available and used without further purification. FT-IR spectra were recorded on Perkin Elmer Spectrum GX FT-IR spectrometer using the KBr pellet technique. Electron paramagnetic resonance spectra were recorded at X-band frequencies with a JES—FA200 ESR Spectrometer. Magnetic measurements were performed on a Vibrating sample magnetometer (VSM) at room temperature. The crystal structure of the nickel complex of sulfadiazine was determined using Bruker Kappa Apex-II CCD-4 diffractometer using graphite-monochromatic MoKα radiation at room temperature (293 K).

### Preparation of Ni-SDZ complex

The complex was synthesized using the reflux method at 60–70 °C. The aqueous nickel acetate (1 mmol) solution was added dropwise to a methanol–water solution of sodium salt of sulfadiazine (2 mmol) with constant stirring. The formed precipitate was separated from the solution by filtration, washed, and dried in a desiccator over CaCl_2_. The complex was insoluble in water and in most of the common organic solvents but soluble in dimethyl sulfoxide (DMSO) and dimethylformamide (DMF). The solid precipitates were dissolved in 3-methyl pyridine and left to crystallize. After four weeks, greenish square crystals were obtained and picked up for single-crystal X-ray diffraction. Light Green solid yield (70%).

**(SDZ ligand) FT-IR (cm**^**−1**^**)**: 3103 (NH), 3423 ν_as_(NH_2_), 3355 ν_s_(NH_2_), 1652 δ(NH_2_), 1580, 1490 ν-phenyl ring, 1325, 1262 (SO_2_)_as_, 1156 (SO_2_)_sy_, 996, 942 ν(S–N). **(Ni-SDZ complex) FT-IR (cm**^**−1**^**)**: 3438 ν_as_(NH_2_), 3299 ν_s_(NH_2_), 1628 δ(NH_2_), 1583, 1598 ν-phenyl ring, 1354, 1274 (SO_2_)_as_, 1179 (SO_2_)_sy_, 1016, 990 ν(S–N), 481 (M–N)^1^. UV–Vis: λ_max_ (DMSO) nm: 259.

### X-ray diffraction analysis

Diffraction data were collected on a Bruker Kappa Apex-II CCD-4 diffractometer using graphite-monochromatic MoKα radiation (0.71073 Å) at 293(2) K^39^. The good quality of the crystal is reflected by the comparatively low value of the R_int_ parameter. The structure is solved by direct methods (SHELXS-97) and refined by full-matrix least-squares methods on |F|^2^ with all non-hydrogen atoms anisotropic (SHELXL-97)^40^. Hydrogen atoms are located from difference maps and refined isotropically. The calculations are performed using the WinGX crystallographic software package^41^. Crystal data and structure refinement results for the compound are summarized in Table 1. The pictures are made with ORTEP-III^42^. Crystallographic data in CIF format are deposited in the Cambridge Crystallographic Data Center, CCDC No. 1552740.

**Table 1:** Preliminary crystallographic data and refinement parameters.

Chemical name	Nickel (4-amino-N-pyrimidin- 2-yl-benzenesulfonamide) (3-methyl Pyridine)
Chemical formula	Ni_0.5_ (C_10_H_10_N_4_O_2_S) (C_6_H_7_N)
Molecular weight	743.5 amu
Crystal system	monoclinic
Space group	P2_1_/c
*a*	8.8364 (10)Å
*b*	18.27001 (20) Å
*c*	13.7006(12)Å
*β*	121.753 (5)
Volume (V)	1881.21 (69) Å3
Z	2
ρ_c_ (Mg/cm^3^)	1.31
µ mm^-1^	0.68
F(000)	772
Size of the crystal	0.34 × 0.15 × 0.05 mm
θ range for data collection (°)	θ_min_ = 2.1°, θ_max_ = 25.0°
Total number of measured reflections	9258
Total number of independent reflections	3315
*h*	− 10 to 10
*k*	− 11 to 21
*l*	− 15 to 16
Number of parameters	224
Goodness of fit on |F|^2^	1.2

### Computational analysis

The Hirshfeld surfaces of the molecule are generated by Crystal Explorer 3.1^43^. The two-dimensional fingerprint plot for Ni-SDZ molecule, SDZ, and 3-methyl pyridine (Solvent) displays the contributions from different contacts: All, H…H, O…H, C…H and N…H, which investigates the intermolecular contacts and gives a quantitative summary of nature and type of intermolecular contacts qualified by the molecules in the crystal. The fingerprint plot is based on the d_e_ (distance from the point to the nearest nucleus external to the surface) and d_i_ (the distance to the nearest nucleus internal to the surface). The value of dnorm is depending on the intermolecular contacts being less than, greater than, and equal to the van der Waals separations and is shown by the surface with a red spot representing shorter contacts, white areas representing contacts around the van der Waals separation, and blue regions are devoid of close contacts. The surface shape index and curvedness highlighted the C–H…π and π…π intermolecular interactions respectively^44,45^. All the calculations of an optimized structure of Ni-SDZ molecule were performed using the Jaguar program as built in the Schrödinger package software at B3LYP/LAV2P* level of theory^46–50^. The wide energy difference between Homo and Lumo is pointing towards the stability of the molecule. Natural bond orbital (NBO) calculation program incorporated in Schrödinger software package.

### ADMET-SAR parameter

The pharmacokinetic properties, such as Absorption, Distribution, Metabolism, Excretion, and Toxicity (ADMET) of the compound were predicted using the preadmet. This tool is a convenient platform for biochemical and drug discovery research investigations. Various chemical-physical characteristics of the compound along with ADMET and toxicity were predicted^51^.

### DNA interaction studies

The stock solution of DNA-polymerized CT-DNA was prepared by dissolving DNA in 10 mM Tris–HCl buffer pH 7.4 and kept for 24 h. The DNA solution was stored at 4 °C and used within 5 days of preparation. CT-DNA solutions gave a ratio of UV absorbance ~ 1.8 at 260 and 280 nm, indicating that DNA was sufficiently free from protein. The nucleotide concentration was determined by UV absorption spectroscopy using the molar absorption coefficient (ε = 6600 M^−1^ cm^−1^) at 260 nm. The ability of the Ni-SDZ complex to interact with CT-DNA was studied by UV–Vis. absorption titration and viscosity measurements according to the literature procedure^52,53^. The absorption spectra of the Ni-SDZ complex were recorded between 200 and 800 nm. The UV–Vis absorption titrations were carried out with a constant concentration of the test complex (5.0 × 10^–5^ M) and various concentrations of CT-DNA (0 to 2.5 × 10^–5^ M). Viscosity measurement was performed in the thermostatic viscosity bath maintained at 37 °C using Ubbelohde viscometer by measuring the flow time of DNA in Tris–HCl buffer, pH 7.2. The flow time of each sample was automatically measured with three repetitions and the viscosity of the samples was computed by the average values of three measurements. The results were presented as relative specific viscosity (η/η_0_)^1/3^ against^19^/[DNA] = 0, 0.4, 0.8, 1.2, 1.6, 2.0, where η and η_0_ are the specific viscosities of DNA in the presence and absence of the compound respectively^54–58^.

### Cytotoxic studies

The cellular level bioassay was carried out using *S. pombe* cells. The cells were grown in 50 mL yeast extract media in a 150 mL Erlenmeyer flask. The flask was incubated at 30 °C on a shaker at 150 rpm till the exponential growth of *S. pombe* was obtained (24–30 h). The cell culture was treated with different concentrations (2, 4, 6, 8, 10 mg mL^−1^) of complexes, free ligands, and DMSO (control) and incubated for 16–18 h. Brine shrimp lethality assay is an important tool for the preliminary cytotoxicity assay based on the ability to kill a laboratory-cultured larva (nauplii). Experiments were concluded with control and different concentration of the complexes. The whole set was triplicated to get an accurate result. The LC_50_ was determined from the best-fit line, a graph of % mortality against the concentration. The LC_50_ value was obtained from the antilogarithm of log^19^ at 50% mortality (LC_50_). All data were collected from three independent experiments^59–61^.

### Photocatalytic degradation of MB

The photocatalytic activities of Ni-SDZ complex-based photocatalysts were calculated by the photodegradation of MB dye under a 24 W lamp irradiation (visible light) in the open air and at room temperature. The distance between the light source and the beaker containing the reaction mixture was fixed at 3 cm. The Ni-SDZ (0.1 g) metal complex photocatalysts were dispersed into 100.0 ml MB (10 PPM) aqueous solutions. Before irradiation (visible light), all three suspensions were magnetically stirred in the dark for 10-min in acetone to ensure the establishment of equilibrium and then were heated to evaporate the acetone until the three separately prepared compounds were deposited in the bottom of the beaker. Samples were withdrawn at regular intervals (20 min) for UV analysis.

## Results and discussion

### Crystal structure of [Ni(SDZ)_2_(3-methyl pyridine)_2_]

**Figure 1:**
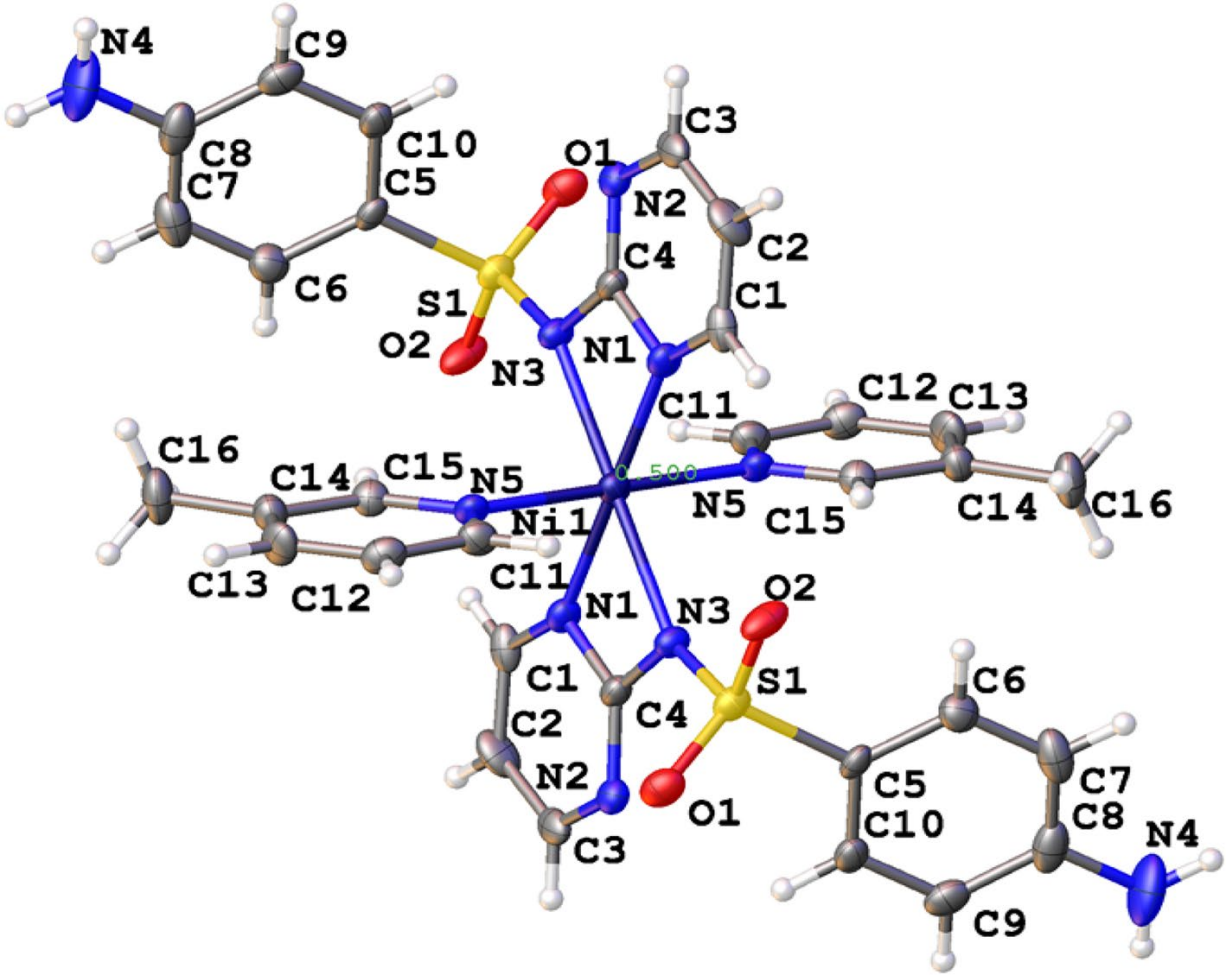
ORTEP View of Ni-SDZ molecule showing the numbering scheme of their displacement ellipsoids at the 50% probability level.

The crystal structure of the Ni-SDZ molecule and the atom numbering scheme are shown in Fig. 1. The preliminary and intensity data of crystallography are given in Table 1. The Ni(II) ion in the Ni-SDZ molecule exhibits a distorted-octahedral environment. The primary ligand sulfadiazine (SDZ) acts as a bidentate sulfonamide ligand and it is coordinated with the sulfonamide N1 and N3 [Ni–N(1) = 2.073(5)Å; Ni–N(3) = 2.145(5)Å]. The apical position is occupied by a 3-methyl pyridine solvent molecule that coordinates metal and its bond distance surrounding the nickel atom is Ni–N(5) = 2.078(5) Å, and the molecule completes the coordination sphere. In the Ni-SDZ molecule, the bond length of the benzene ring is slightly greater than the free SDZ ligand**,** whereas distances S(1)–N(1) = 1.614(5) Å, is slightly less than the free SDZ ligand. In the reported molecule (Ni-SDZ), bond length of S1–O1 = 1.441(5) Å and S1–O2 = 1.446(4) Å is slightly greater than the free SDZ ligand. Also, the bond angle of O1–S1–O2, O1–S1–C5, O2–S1–C5, and C5–S1–N3 is nearly equal to the free SDZ ligand^62^. The selected bond parameters surrounding the Ni(II) in [Ni(SDZ)(3-methyl pyridine)] with experimental (X-ray) and Computational (B3LYP) data are reported in Table 2.

**Table 2:** Bond lengths (Å) and angles (°) surrounding the Ni(II) in Ni-SDZ molecule with experimental (X-ray) and theoretical (B3LYP) data.

Atom	X-ray	B3LYP	Atom	X-ray	B3LYP
Ni1—N1	2.073 (5)	2.123	N1—Ni1—N5	91.2 (2)	89.878
Ni1—N5	2.078 (5)	2.099	N1—Ni1—N3	63.63 (18)	62.653
Ni1—N3	2.145 (5)	2.193	N5—Ni1—N3	90.6 (19)	91.958

**Figure 2:**
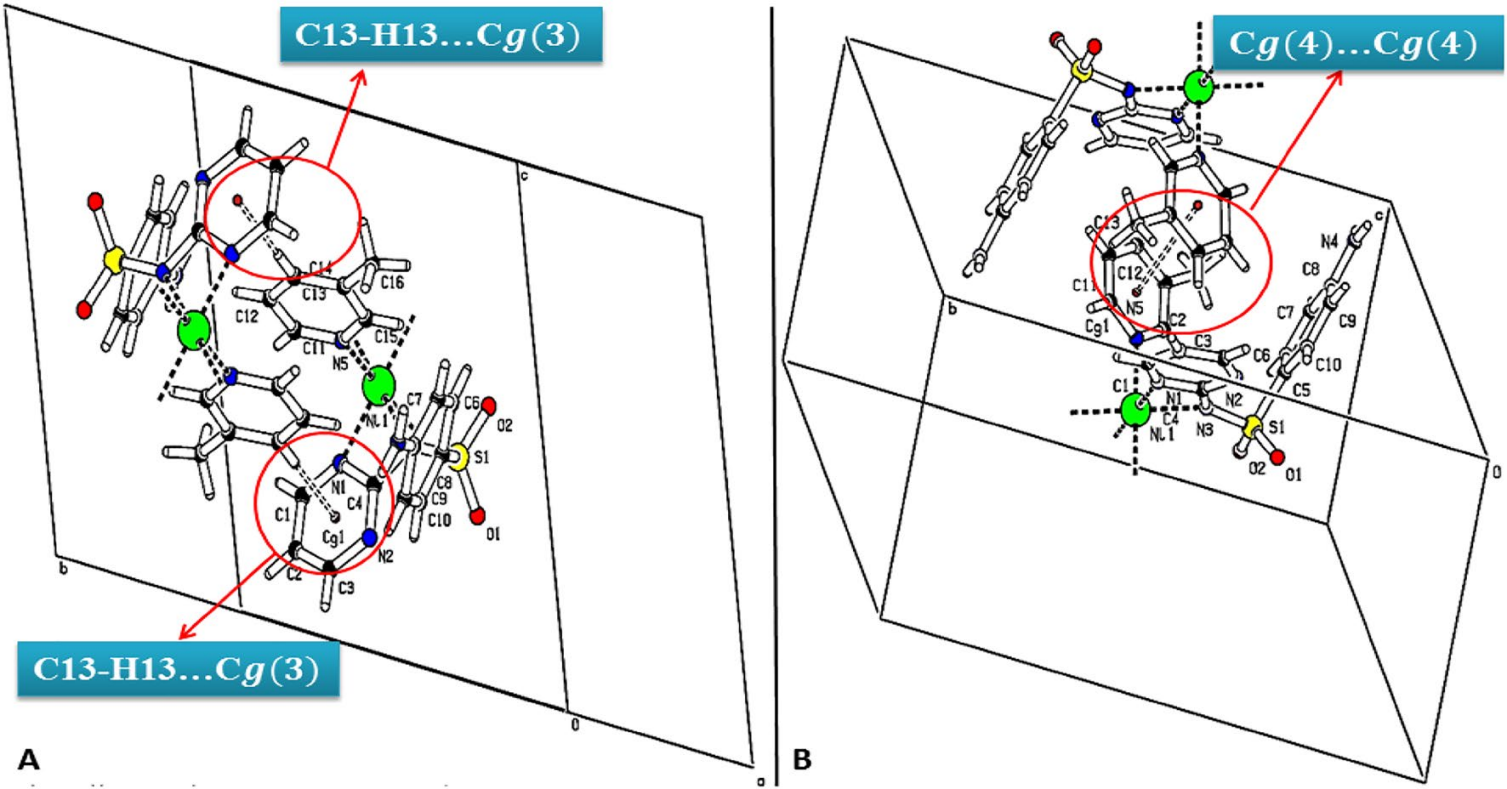
Crystal packing of Ni-SDZ molecule showing (**A**) C–H···π and (**B**) π…π Interactions.

**Figure 3:**
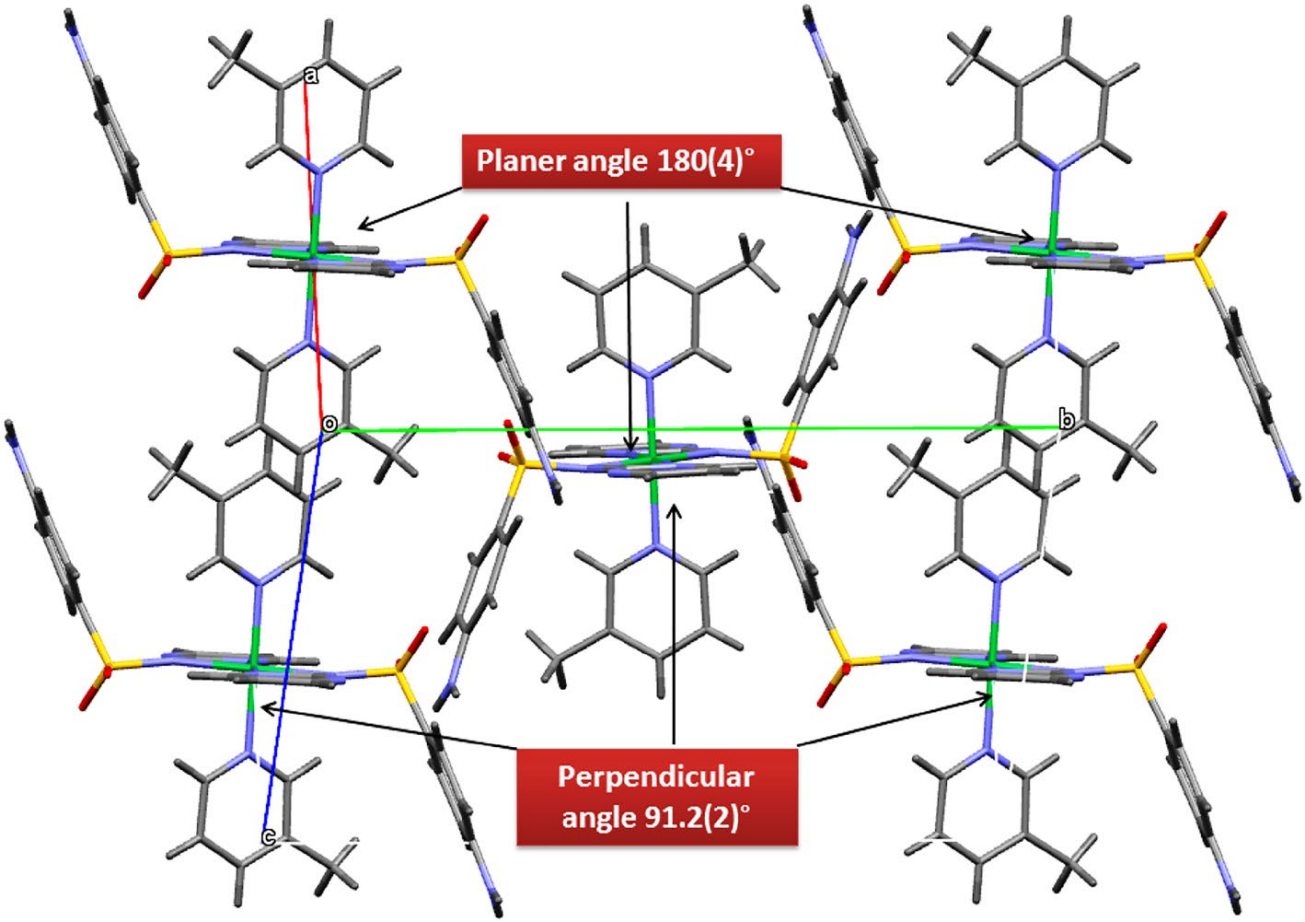
Packing diagram of Ni-SDZ molecule containing an orthogonal view of ligand (sulfonamide) and solvent (3-methyl pyridine).

The stereochemistry of the sulfur atom is as usual a slightly distorted tetrahedral geometry with the bond parameters involving sulfur lying within the range quoted in the literature^63,64^. The maximum and minimum values of angles around the sulfur atom are O1–S1–O2 and O2–S1–N3 116.6(3) Å and 105.2(3) Å respectively for Ni-SDZ and the S–N bond length is similar to that found in reported structure [Ni(C_10_H_9_N_4_O_2_S)_2_(C_5_H_5_N)_2_]·0.5H_2_O^65^. Intramolecular and intermolecular interactions play a vital role in the stability of crystals. Few intermolecular interactions like Π…Π [Cg(4)–Cg(4)] and C–H…Π [C(13)–H(13)–Cg(3)] also help in the stability of the molecule and their relevant distance are 3.791 Å and 2.57 Å respectively. The C–H…Π [C(13)–H(13)–Cg(3)] interaction distance (2.57 Å) in the present molecule which is quite lesser than the distance in the reported crystal structure (C–H–Cg distance of 3.605(7) Å)^66^. The C–H…Π and Π…Π Interactions are presented in Fig. 2. The molecular structure shows the heterocyclic ring is planar. The nickel is in a similar plane as the four coordination 3-methyl pyridine nitrogen. The angles to the least square plane through nickel, sulfonamide, and heterocyclic nitrogen [N1–Ni–N1^i^] are 1.2(4)°, and 180(4)° respectively and the angle between sulfonamide and 3-methyl pyridine nitrogen is almost perpendicular [N1–Ni1–N5 = 91.2(2)°] (Fig. 3).

### FT-IR spectral analysis

To clarify the mode of bonding and the effect of the nickel ion on the SDZ ligand, the IR spectra of the SDZ ligand and their nickel complex are calculated and assigned based on a careful comparison of their spectra with that of the SDZ ligand. The infrared spectra (FT-IR) of the nickel complex taken in the region 4000–400 cm^-1^ are compared with the SDZ ligand. Based on some general references^33,34^ and earlier studies of complexes with sulfonamide derivatives^67–70^, a tentative assignment of the best important bands is given in Figure S2. The symmetric and asymmetric bands assigned to ν(NH_2_) in SDZ ligand (3496 and 3317 cm^−1^) are shifted to wave numbers in the nickel complex (3490 and 3382 cm^−1^), representing the NH_2_ group are modified to the free SDZ ligand. These modifications are probably owing to the hydrogen bonding between complexes involving the NH_2_ and SO_2_ groups. The scissoring vibrations for the amino (–NH_2_) group seem at 1625 cm^−1^ and the peak due to the phenyl ring is at 1530 cm^−1^. The peaks at 1339 cm^−1^assigned to ν_asym_(SO_2_) and those at 1154 cm^−1^ to ν_sym_(SO_2_) show important changes upon complexation. The 1066 cm^−1^ band in the ligand is assigned to ν(S–N)^71^ at higher frequencies. These shifts to higher frequencies are in accord with the shortening of the S–N bond lengths which have been observed in the respective crystal structure of the complex. Also, the presence of 406 cm^−1^ in Ni-SDZ spectra indicates metal–ligand binding which is absent in the spectra of SDZ ligand^60,72^.

### Magnetic behavior studies

**Figure 4:**
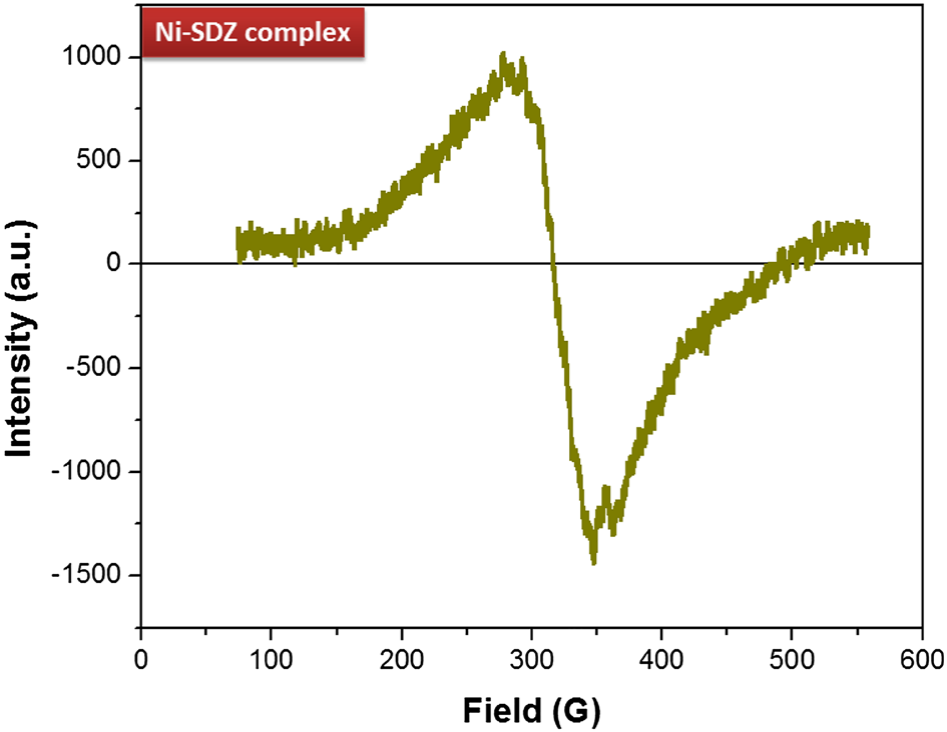
Electron paramagnetic resonance spectra (EPR) of nickel complex of SDZ.

The room temperature X-band EPR spectrum of the nickel complex is shown in Fig. 4. The EPR spectrum of the Ni-SDZ complex is exhibiting both parallel and perpendicular g tensor values which are 2.371 for g_parallel_ and 1.942 for g_perpendicular_. The experimental g values were g_parallel_ > g_perpendicular_ > 2.0023 which indicates that the nickel has a ground state characteristic for octahedral or square planar geometry. Also, we compute the g_avg_ = 2.085 with the help of equation g^2^_avg_ = 1/3[2g^2^_perpendicular_ + g^2^_parallel_]. The g_parallel_ > g_perpendicular_ value suggests the octahedral environment of the nickel complex of SDZ^73,74^.

The magnetic behavior of the complex was studied at room temperature (293 K) (Figure S3). The summary of the results on the magnetic behaviour of the nickel complex was given by Figgis and Nyholm^75^. The experimental values of the magnetic moment for nickel complexes are generally revealing of the coordination geometry of the metal. Nickel must exhibit a magnetic moment higher than that expected for two unpaired electrons in octahedral (2.80–3.20 B.M) and tetrahedral (3.40–4.20 B.M.) complexes, while its square planar complexes would be diamagnetic. The magnetic moment observed for the nickel(II) complex is 2.83 B.M which is consistent with the octahedral stereochemistry of the complex. Also, The nature of the graph of magnetic susceptibility: χ = M/H with a slope of 2.227 × 10^–7^, reveals that the complex possesses paramagnetic properties.

### Hirshfeld surface analysis

**Figure 5:**
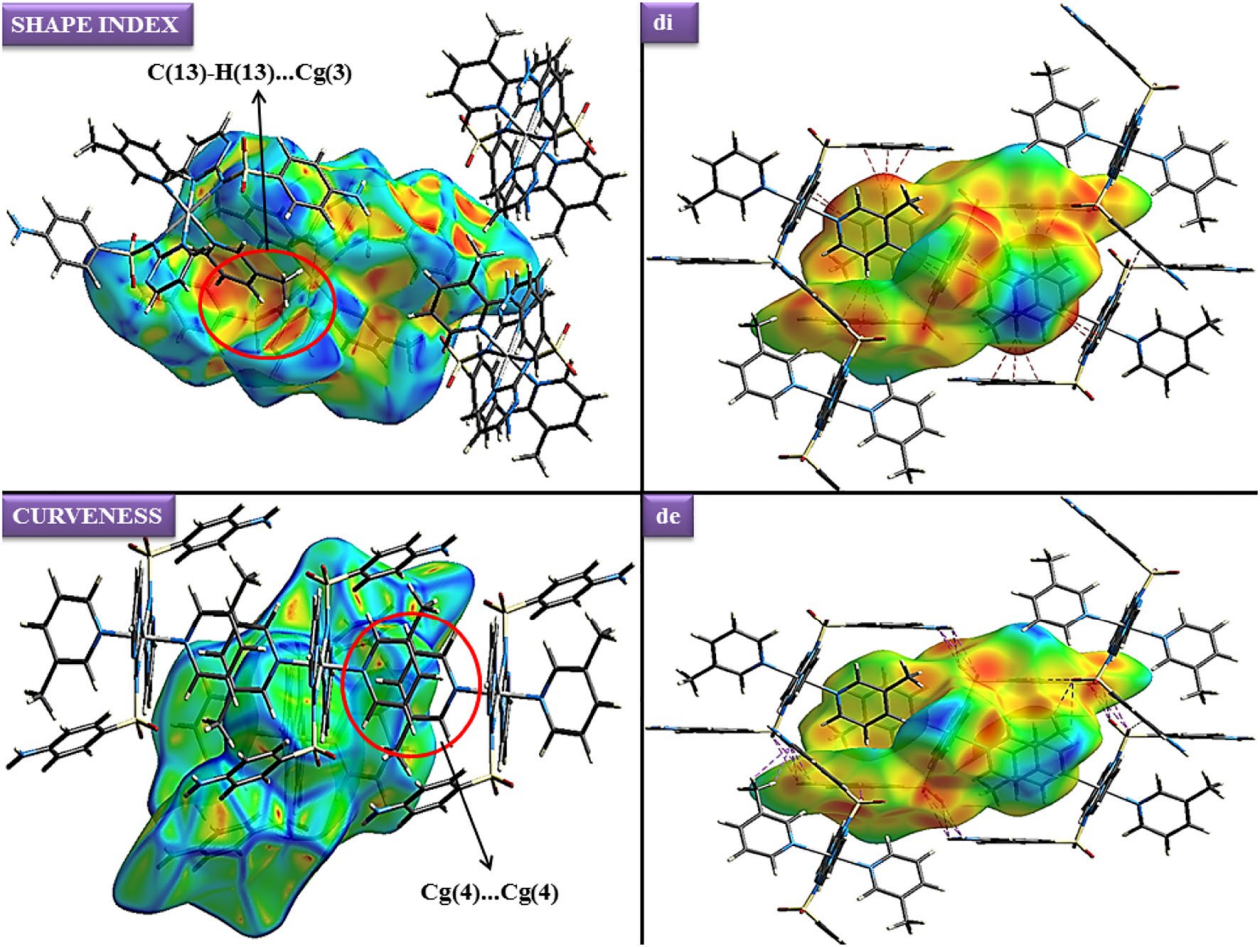
Hirshfeld surfaces of Ni-SDZ molecule showing shape index, curvedness, di, and de surface property.

**Figure 6:**
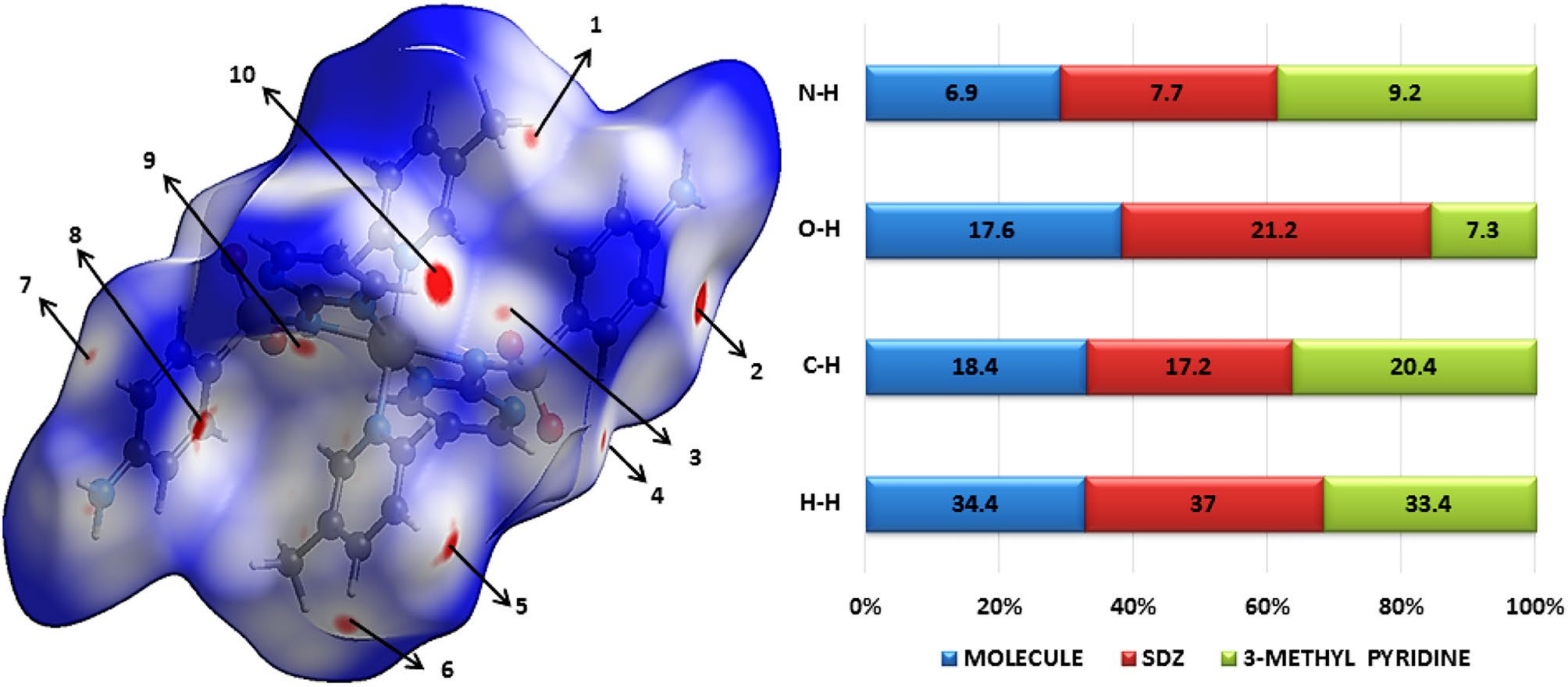
Hirshfeld surface is visualizing dnorm surface and the column chart shows the percentage contributions of various contacts of molecule Ni-SDZ molecule, SDZ, and 3-methyl pyridine (Solvent).

**Figure 7:**
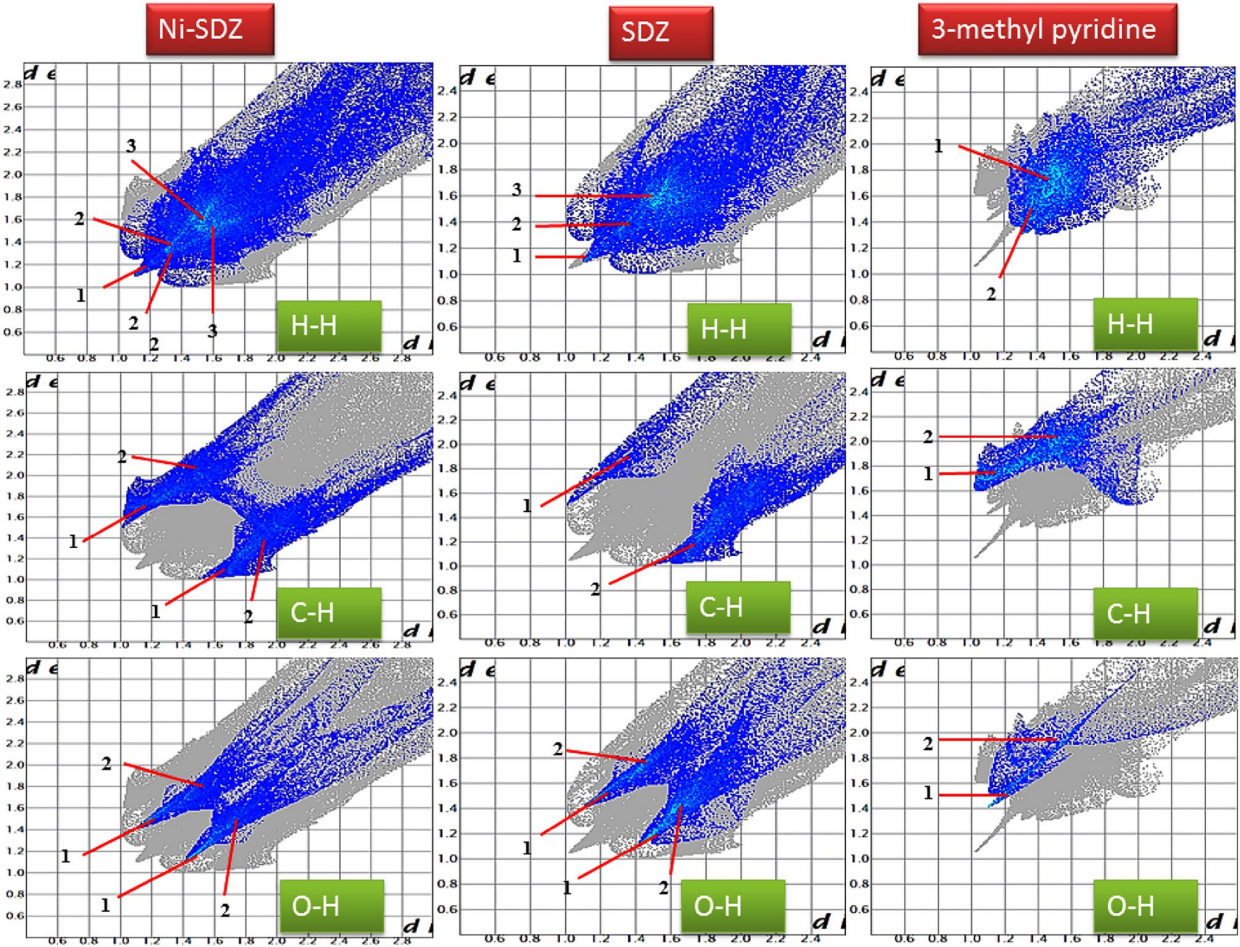
2-D fingerprint plot of Ni-SDZ molecule, SDZ and 3-methyl pyridine (Solvent).

Hirshfeld surface analysis and their correlated 2D fingerprint plots^76^ have been performed to study the nature of the intermolecular interactions and their measurable contributions towards the crystal packing. The Hirshfeld surfaces (shape index, curvedness, di, and de) of the Ni-SDZ molecule are depicted in Fig. 5. The shape index and curvedness plots are significant indicators for the C–H…π interactions and π…π stacking within the crystal lattice^77^. There are touching pair of triangles (blue and red) for the shape index and the curvedness surfaces display broad, relatively flat regions characteristic of π-stacking of molecules. One of the significant interactions in the molecule is between C…H/H…C atoms with the distances of 1.08 Å and 1.45 Å for H to di surface and C to di surface respectively and so, the total distance of C…H/H…C interactions is 2.53 Å. The dominant interactions N–H…O and C–H…O is observed on the de surface. The distance of O…H (N–H…O) is 2.55 Å and the distances of O to de and de to H are 1.438 Å and 1.11 Å respectively. The normalized contact distance (dnorm) is based on both de and di. The surface represented by a deep red spot is illuminating hydrogen bonding interactions (Fig. 5)^58,78^. The dominant interactions are H…O can be seen in di and de surface plots as the bright red spots and deep red spots in maps are showing strong interactions in molecules. The column chart shows the percentage contributions of various contacts of the molecule (Ni-SDZ molecule), SDZ, and 3-methyl pyridine (Solvent) in Fig. 6. The red spot in the dnorm surface (Fig. 6) is attributed to weak hydrogen bonds involving the acceptor oxygen and nitrogen atom from the sulfonyl group and the hydrogen atom from the pyrimidinyl ring. The 2D fingerprint plots can be deconstructed to focus on particular atom pair contacts. At the top left and bottom right of the fingerprint plot, there are characteristic ‘‘wings’’ which are identified as a result of C…H contacts and O…H interactions are represented by a spike (Fig. 7). The 2D fingerprint plots of the Ni-SDZ molecule, SDZ, and 3-methyl pyridine shows a variety of contacts (H…H, C…H, O…H and N…H) in the crystal structure. The percentages contributions of H…H (37%) of SDZ is higher than Ni-SDZ and 3-methyl pyridine and the contribution C…H (20.4%) of 3-methyl pyridine is higher than Ni-SDZ molecule (18.4%) and SDZ (17.2%). The Hirshfeld surfaces certainly allow a detailed analysis by displaying all the intermolecular interactions within the Ni-SDZ molecule, SDZ, and 3-methyl pyridine^77,79^.

### Computational analysis

#### Optimized geometry

An optimized molecular structure of the Ni-SDZ is calculated at B3LYP/LAV2P level by using the Jaguar program in Schrödinger software^80^. The experimental data are in a crystalline state whereas the Computational data are in a gaseous state. Optimized bond lengths and bond angles are listed in Table 2 and correlation coefficient values for molecular geometry (bond length and bond angle) are 0.982 and 0.989 respectively and the results are represented graphically (Figure S 4).

#### Mullikan population analysis (MPA)

**Figure 8:**
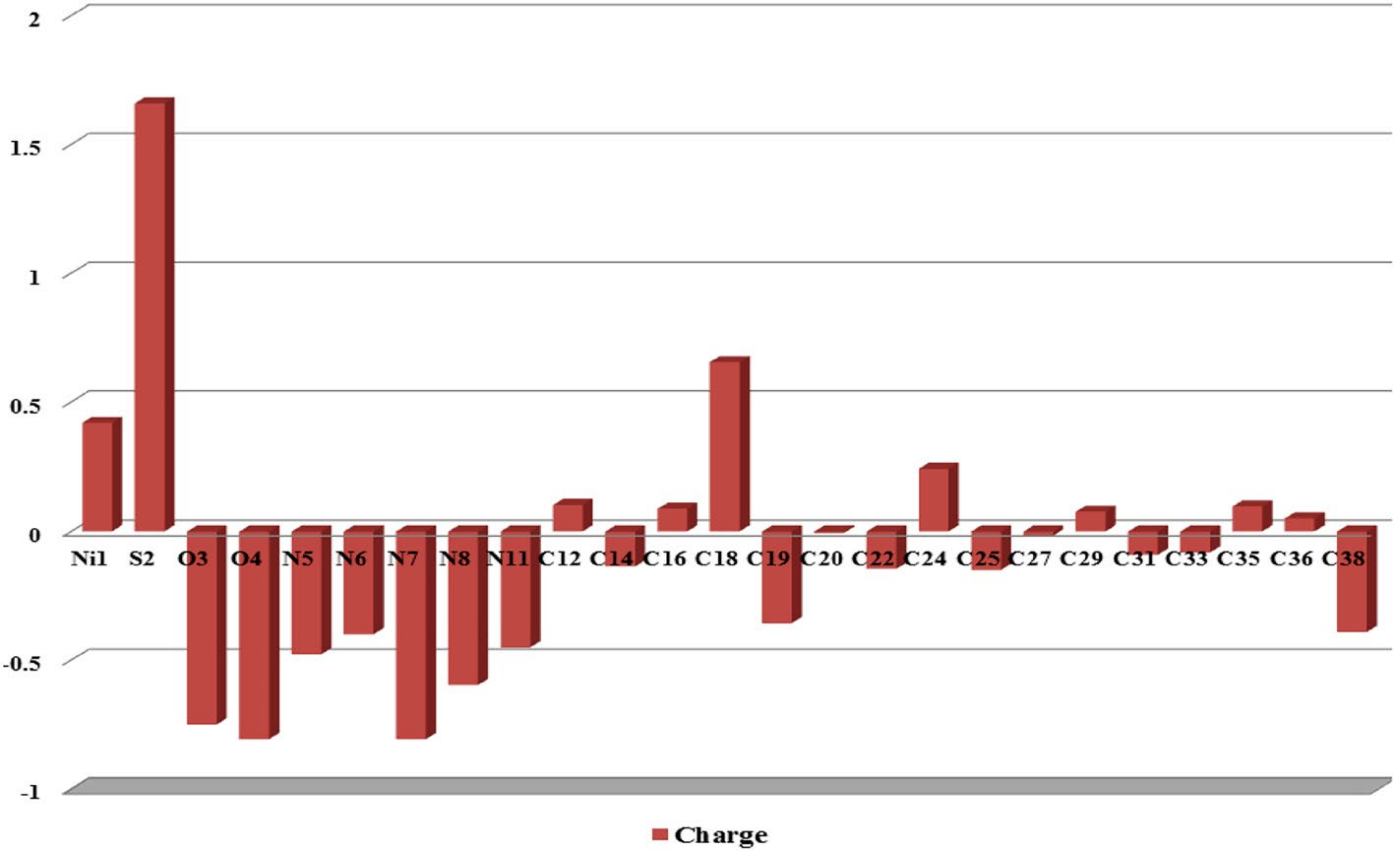
Histogram for Mulliken atomic charges of Ni-SDZ molecule.

Mullikan population analysis (MPA)^46^ of the Ni-SDZ is performed at B3LYP/6-31G(d,p) level to obtain the values of the atomic charges. The bonding capability of a molecule depends on the electronic charge of the atoms. The calculated Mulliken charge values are listed in Table S1 and represented graphical forms of our results were shown in Fig. 8. The investigation expresses that the atom N7 shows the largest electro negativity (− 0.80577) and S2 shows the largest electro positivity (1.65838) which indicates the large charge delocalization in Ni-SDZ and the positive charges are localized due to hydrogen atoms. The analysis shows the presence of large electronegative atoms O3 (− 0.74922) and O4 (− 0.80557) creates a more positive charge on S2 (1.65838) atom. In the phenyl ring, all the carbon atoms have negative charges except C24 (0.2426). This suggests that atom C24 is acting as the center for charge transfer between the NH_2_ substituent and the phenyl ring^81^.

#### HOMO–LUMO energy

**Figure 9:**
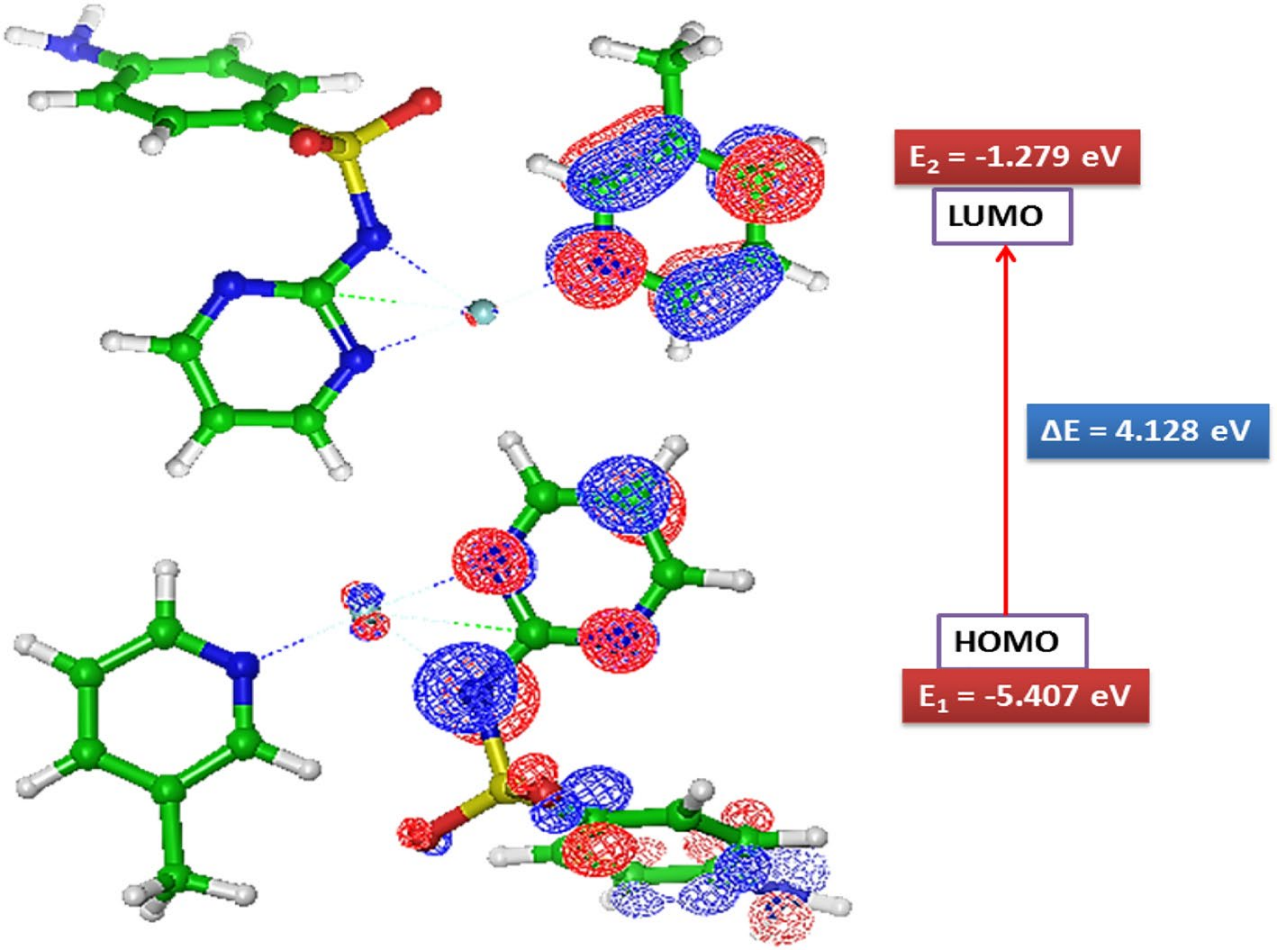
Homo–Lumo energy of Ni-SDZ Molecule.

Homo and Lumo energy calculated by B3LYP/LAV2P* method is depicted in Fig. 9 Homo (− 5.407 eV) and Lumo (− 1.279 eV) energy represents an ability to donate and accept the electron. The electronic absorption agrees with the transition from the ground to the first excited state which is largely described by one electron excitation from the highest occupied molecular orbital to the lowest unoccupied molecular orbital^46,82^. The wide energy difference between Homo and Lumo points towards the stability of the molecules and this energy gap (4.128 eV) reflects the chemical activity of the molecule. The UV–Vis spectra report the absorption maxima (265 nm) and optical energy band gap (4.683 eV). The energy gap between the HOMO and LUMO orbital has been found 4.128 eV as calculated theoretically. The computational (HOMO–LUMO) and experimental (UV–Vis) energy gap is measured in different conditions of the environment, therefore smaller deviation has been observed in it^78^.

By using Homo and Lumo energy values of a molecule, the global chemical reactivity descriptor of molecules such as Electronegativity (χ), global hardness (η), global softness (S), and electrophilicity (ω) are calculated using Koopman’s theorem^83^. Electronegativity (3.343 eV) is a chemical property that describes the ability of an atom to attract electrons or electron density toward itself. The Global hardness (2.064 eV) is a measure of the resistance of an atom to charge transfer, which indicates the molecule is more reactive and less stable. The global softness (0.4845 eV) which is equal to the reciprocal of global hardness describes the capacity of an atom or a functional group to attract electrons. The quantity global electrophilic power of the compound is electrophilicity (2.7205 eV) which defines a quantitative classification of the global electrophilic nature of a compound and measure of energy lowering due to maximal electron flow between donor and acceptor^84^.

#### Natural bond orbital analysis (NBO)

Natural bond orbital (NBO) analysis is useful to investigate the stability of the molecule (Ni-SDZ) arising from charge delocalization. NBO analysis provides an essential method for understanding the interaction among bonds that played a vital role in the stabilization of the molecule. The second-order Fock matrix has been carried out to evaluate the donor–acceptor interactions in the NBO analysis. E^(2)^ which represents the stabilization energy associated with i(donor)–j(acceptor) delocalization is estimated from the second-order perturbation approach. For each donor NBO (L) and acceptor NBO (NL), the stabilization energy E^(2)^ associated with delocalization is given as:

$$ {\text{E}}^{{({2})}} = {\text{E}}_{{{\text{NL}},{\text{L}}}} = {\text{q}}_{{\text{l}}} \left[ {{\text{F}}\left( {{\text{L}},{\text{NL}}} \right)/{\text{E}}\left( {{\text{NL}}} \right) - {\text{E}}\left( {\text{L}} \right)} \right] $$

where q_l_ is the donor orbital occupancy, E(NL) and E(L) are diagonal elements (orbital energies) and F(L, NL) is the off-diagonal NBO Fock matrix element. The higher value of E^(2)^ shows the more intense the interaction between electron donors and electron acceptors^46^. The LP(2) N7(donor), LV(1) C18(acceptor) interactions have the highest E^(2)^ value of 131.55 kcal/mol. The donor and acceptor orbitals played the most dominant role in stabilizing the molecule (Table 3). The charge transfer interaction has been formed by the orbital overlap between bonding and antibonding orbital. The types of charge transfer in molecules are found to be n → σ ∗ , n → π ∗ and π → π ∗ . The co-ordination covalent bond of non-lewis nickel(acceptor) with lewis type of one 3-methyl pyridine (solvent) nitrogen and two sulfonamide nitrogen (Ni–N) have the E^(2)^ values of 17.65, 7.08, and 16.57 kcal/mol respectively.

**Table 3:** Second-order perturbation theory analysis of Fock matrix in NBO basis for Ni-SDZ molecule.

Donor (L) NBO	Acceptor (NL) NBO	E^(2)^ kcal/mol	E(NL)–E(L) a.u
LP (1) N5	LV (6) Ni1	17.65	0.6
LP (2) O3	σ* S2–C19	7.68	0.45
LP (3) O3	σ* S2–O4	8.98	0.5
LP (3) O3	σ* S2–N7	6.94	0.41
LP (2) O4	σ* S2–C19	6.26	0.45
LP (3) O4	σ* S2–O3	9.18	0.51
LP (1) N6	σ* N5–C18	7.08	0.79
LP (2) N7	LV (1) C18	131.55	0.09
LP (1) N8	π* C22–C24	16.57	0.32
LP (1) C14	π*N5–C12	84.02	0.11
LP (1) C14	π*N6–C16	71.37	0.13
π N5–C12	LV (1) C18	32.72	0.2
π N6–C16	LV (1) C18	44.31	0.18
π C19–C20	π* C22–C24	7.77	0.28
π C19–C20	π*C25–C27	12.15	0.29
π C22–C24	π* C19–C20	14.47	0.27
π C22–C24	π* C 25–C27	7.68	0.29
π C25–C27	π* C19–C20	8.4	0.27
π C25–C27	π* C22–C24	12.69	0.27
LP (1) N11	LV (6)Ni1	20.36	0.62
π N11–C 29	π* C35–C36	11.38	0.35
π C 31–C 33	π*N11–C29	16.48	0.24
π C 31–C 33	π*C35–C36	8.61	0.3
π C 35–C 36	π* N11–C29	9.46	0.23
π C 35–C 36	π* C31–C33	12.36	0.27

### ADMET-SAR parameters

The Caco-2 cellular permeability of the chemical was predicted by the absorption (A) study to be medium. The results in Table 4 indicate that the compound's capability to bind to plasma proteins was modest. Usually, P-glycoprotein binders facilitate the excretion of chemicals from the cells through the drug efflux mechanism and increase drug resistance. The results revealed that the compound was neither a substrate nor an inhibitor of P-glycoprotein. Human Intestinal Absorbance data revealed that the compound has the potential to be well absorbed. The compound has lower MDCK permeability. Drug distribution (D) analysis predicted to the compound cannot cross the blood–brain barrier (BBB). In the analysis of liver microsomal metabolism, the 2 key cytochrome P450s (CYP450s) which are involved in drug metabolism are CYP2D6 and CYP3A4; these two enzymes together perform the majority of the liver microsomal phase-I drug metabolism. The results showed that the compound was an inhibitor of CYP2D6 and CYP3A4; not a substrate for CYP3A4 and CYP2D6. Toxicity parameters of the compound reflected that it has a medium risk for hERG inhibition^85^.

**Table 4:** Computational studies of a molecule.

*ADMET parameters*			
BBB	0.0355242	Skin permeability	− 4.15228
Caco2	17.8992	CYP 2C19 inhibition	Non
HIA	94.384107	CYP_2C9 inhibition	Non
MDCK	0.518548	CYP_2D6 inhibition	Inhibitor
Pgp inhibition	Non	CYP 3A4 inhibition	Inhibitor
Plasma Protein Binding	48.744991	hERG inhibition	Medium risk

BBB = blood–brain barrier (high absorption CNS > 2.0, middle absorption CNS 2.0–0.1, low absorption to CNS < 0.1, Caco2 (high permeability > 70, middle permeability 4–70, low permeability < 4, %HIA (human intestinal absorbance) (well-absorbed compounds 70–100%, moderately absorbed compounds 20%–70%, poorly absorbed compounds 0–20%), %PPB (plasma protein binding) (strongly bound > 90%, weakly bound < 90%), MDCK (higher permeability > 500, medium permeability 25–500, lower permeability < 25).

### CT-DNA interaction studies

**Figure 10:**
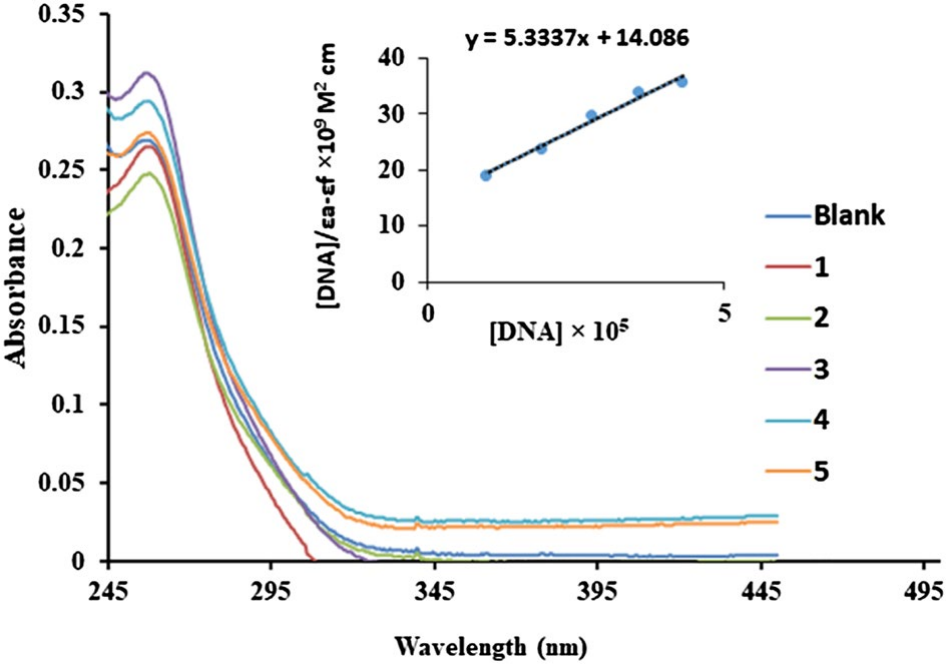
Absorption spectra of complex with increasing amount of CT-DNA in Tris–HCl buffer (pH 7.2)^19^. = 5 µM, [DNA] = 0–60 µM with an incubation period of 10 min at room temperature.

Polycyclic aromatic molecules can interact with DNA either through forming covalent bonding with DNA or by some non-covalent interaction like groove binding, electrostatic interaction, and intercalation. In groove binding, the molecules locate themselves in the major or minor groove of the DNA, and forces like hydrogen bonding, van der Waals, or hydrophobic interaction stabilizes the molecule-DNA conformation. Intercalation occurs when molecules of a reasonable size and chemical nature fit in between the stacks of DNA base pairs through associative π-stacking interaction and these often make a good nucleic acid stain^86^. Some intensively studied DNA intercalators are ethidium bromide, daunomycin, doxorubicin, etc. Doxorubicin and daunorubicin are used in the treatment of some tumors. DNA interaction study is very useful in developing a novel class of antitumor agents. UV–visible absorption spectroscopy and relative viscosity measurement studies were used to determine the binding ability of Ni-complex with DNA. The measurement of change in electronic spectra of the metal complex upon successive addition of DNA gives valuable information about the binding mode and strength. The effect of the stoichiometric addition of calf thymus DNA on the UV–visible absorption spectra of Ni-complex is shown in Fig. 10. The increase in the concentration of CT DNA resulted in a hypochromic with bathochromic shift, which suggests that the complex interacts with DNA most likely through a mode that involves a stacking interaction between the aromatic chromophore and the base pairs of DNA. The columbic force between metal ions and the phosphate of nucleotide could favor these interactions. Using absorption titration measurements, a linear plot of [DNA]/(ε_f_ − ε_a_) versus [DNA] (Fig. 10, inset) is obtained. Assuming all the molecules of complexes were bound with DNA, the experimental K_b_ was obtained by substituting the absorbance into Beer’s law. The K_b_ value derived from the plot for the complex is 3.8 × 10^4^ M^-1^. The bathochromic shift, hypochromic, and large binding constant value obtained for Ni-complex suggests that the binding mode is intercalation^21,87–91^.

The change in relative viscosity of DNA with increasing concentration of any compound is directly associated with the mode of interaction. The intercalation of compounds in between the base pair stacks lengthens the DNA size, and so the viscosity of the DNA solution increases. While the partial intercalation or non-classical interactions can bend the DNA, and hence the size of the DNA chain is reduced and so is the viscosity. So, the relative viscosity measurement results are the least ambiguous in finding the mode of interaction between DNA. The relative viscosity of the DNA solution increases with an increase in the concentration of Ni-complex, which is the result of an increase in the size of the DNA chain due to intercalation (Figure S5). Similar kinds of results are observed in the case of reported metal complexes and classical intercalator, EtBr and the data are supporting the UV–visible absorption titration study^55,87,88,92^.

### Molecular docking study

**Figure 11:**
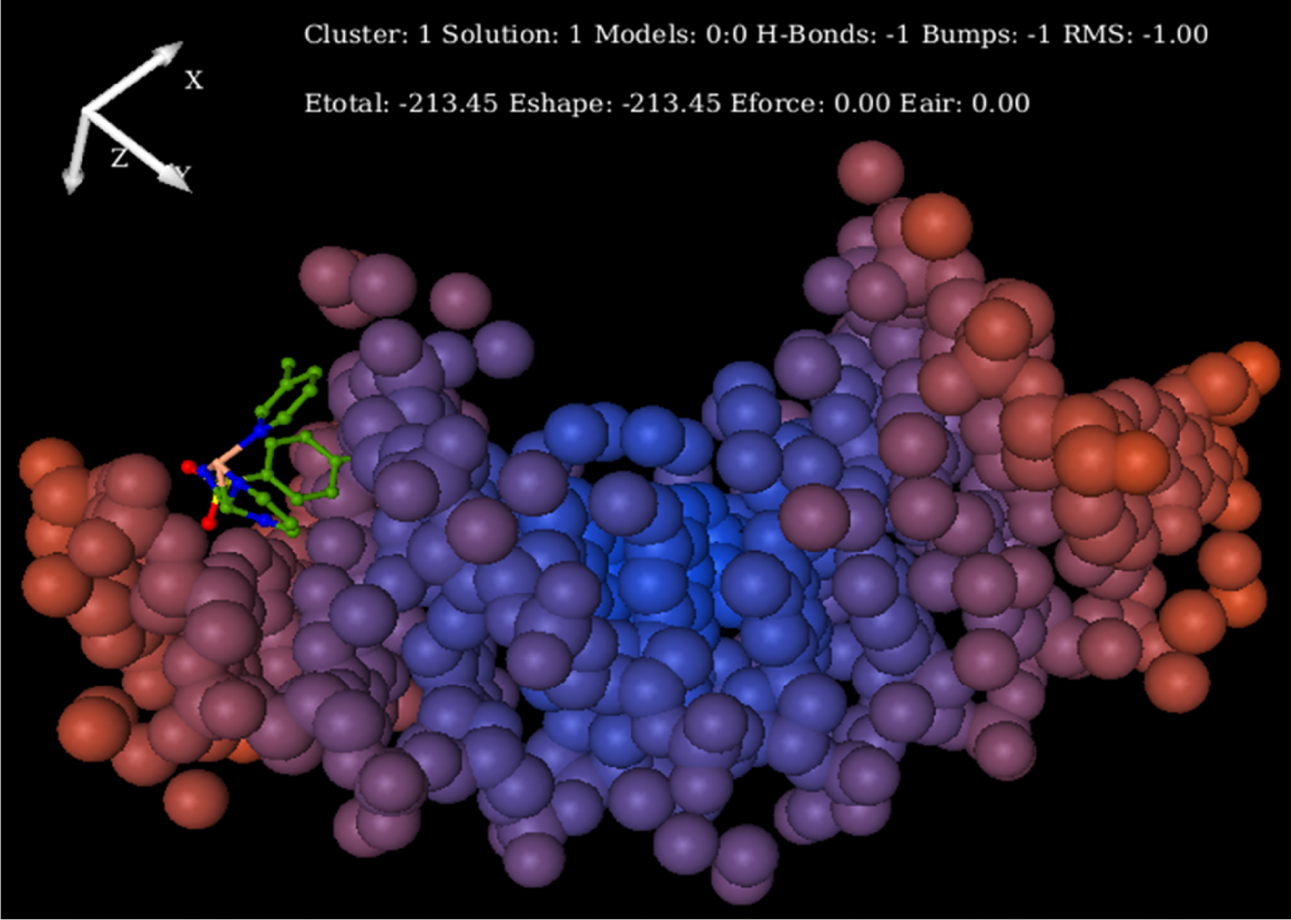
Molecular docked model of a complex located within the hydrophobic pocket of DNA (PDB ID: 1BNA).

Molecular docking is used to predict the structure of intermolecular complexes formed between two or more constituent molecules. Currently, it is actively used in virtual screening, lead optimization, biological activity prediction, binding site identification, drug-biomolecular interaction, etc. So, it has gained enormous interest for researchers as a tool for drug discovery, nowadays. The computational strategy allows for permeating all aspects of drug discovery today. Our interest to find out the primary site of interaction of Ni-complex with DNA was accomplished by molecular docking studies on DNA duplex of self-complementary sequenced (ACCGACGTCGGT)_2_ by Hex software 8.1. The final binding orientation among all possible conformation having optimal energy is considered as a docked pose with minimum energy, which shows that minor groove mode plays a predominant role in the interaction (Fig. 11)^45,93–95^. The relative binding energy of the docked structure was found − 213.45 kJ mol^−1^. The lower binding energy implies a more potent binding affinity between the receptor (DNA) and a metal complex. These data are consistent with the UV–visible absorption titration study and relative viscosity measurement.

### Cytotoxicity and cell viability studies

Cytotoxicity is the most important study among all biological evaluation studies. The compound may have different cytotoxicity mechanisms such as cell membrane destruction, prevention of protein synthesis, irreversible receptor binding, etc. So, the determination of cell death requires cheap, reliable, and reproducible short-term cytotoxicity and cell viability assays, for which a broad spectrum of cytotoxicity assays are currently used. The brine shrimp lethality assay correlates reasonably well with cytotoxic and anti-tumor properties. In the present study, the brine shrimp lethality of the metal complex was determined using the procedure of Meyer et al. The maximum mortality of brine shrimp was observed at a 30 µM concentration of Ni-complex. The LC_50_ value of the metal complex was obtained by a plot of the percentage of the shrimp nauplii killed against the concentrations of the metal complex and the best-fit line was obtained from the data using regression analysis. This significant lethality of the metal complex (LC_50_ = 7.44 µM) to brine shrimp is indicative of the presence of potent cytotoxic components which warrants further investigation.

The proportion of viable cells in a cell population was estimated by the simplest and most widely used dye exclusion method. In the dye exclusion method, viable cells exclude dyes, but dead cells do not exclude them. In vivo*,* cytotoxic study of Ni(II) complex at a cellular level was carried out using eukaryotic *S. pombe* cell by trypan blue assay. The percentage viability of cells treated by complex after 17 h treatment is 92%, 87%, 81%, 79%, and 74%, for series of complex concentrations 2, 4, 6, 8, and 10 µg/mL, respectively. The data clearly shows the potent cytotoxic nature of the complex.

## Photo-catalytic degradation

**Figure 12:**
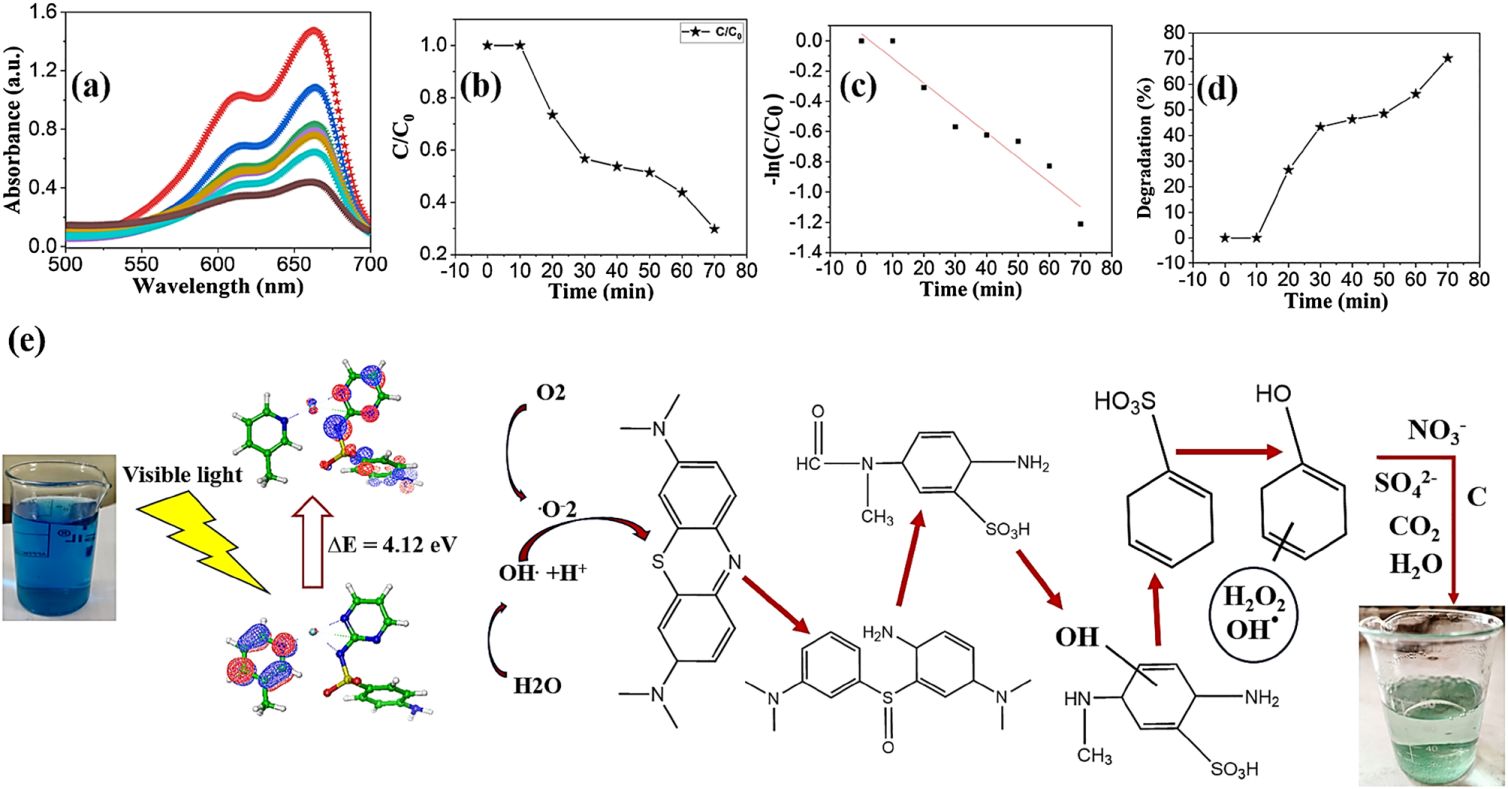
(**a**) Absorption spectra of MB solution with Ni-SDZ complex function of visible light irradiation time, (**b**) Photocatalytic degradation rate of MB in the presence of Ni-SDZ complex, (**c**) The liner fitting of -in C/C_o_ and irradiation time and (**d**) Starting and final degradation % V/S time and (**e**) Degradation pathway of MB dye via Chromophoric group degradation.

The photocatalytic degradation of MB was conducted to investigate the efficiency of photocatalysts. The photocatalytic activities of Ni-SDZ were monitored from the variation of the color in the reaction system by measuring the maximum absorbance intensity of MB. The absorption spectra of a sample (20 min time interval) and MB is shown in Fig. 12a. The absorption spectra of the MB solution decreased with visible light irradiation time. Under visible irradiation in a period of 70 min, the Ni-SDZ complex can degrade over 70.19% of the MB dye (Fig. 12a). The reduction in the intensity of the maximum absorption peak (662 nm) for MB suggests the complete removal of the chromophoric group within the MB molecule (Fig. 12a). If the position of the distinctive peak in the MB absorption spectrum, centered at 662 nm, remains unchanged throughout the experiment despite the diminishing intensity, this signifies the nonexistence of any other chromophore molecules produced as by-products. The complete degradation of MB without any intermediate formation can be examined by the disappearance of the λmax peak (662 nm) without the appearance of other peaks in the UV–vis spectra^96^. At the same time, the photograph of the MB dye degrading sample with time clearly shows the color change of MB dye. The photocatalytic degradation activity of Ni-SDZ complex is more than reported [Co(4,4′‐bipy)∙(HCOO)2]_n_ (54.70% degradation) MOF complex^97^. The curve of changes in the relative concentration of MB dye (calculated from the absorption data) versus visible light irradiation time for each sample is demonstrated in Fig. 12b and the logarithms of the values are shown in Fig. 12c. To have a better understanding of the reaction kinetics of the MB degradation catalyzed by Ni-SDZ photocatalysts, the experimental data were fitted by a first‐order model as expressed by Eq. (1) (Langmuir‐Hinshelwood model) since the value of the rate constant k commonly indicates the activity of the photocatalyst. The degradation pathway starts with electronic reorganization, the sulfhydryl group (C–S^+^=C) transforms a sulfoxide (C–S=O)–C), leading to the opening of the central aromatic heterocycle. The introduction of an electrophilic^.^OH group at the unpaired electron of the S heteroatom results in a change of its oxidation state from − 2 to 0. However, the transition from C–S^+.^C to C–S(^.^O)–C must maintain the preservation of the double bond conjugation, which in turn triggers the unfolding of the central aromatic ring that encompasses both heteroatoms (S and N). The necessary hydrogen atoms for the formation of C–H and N–H bonds may be derived from the reduction of protons due to electrons generated by light. Among the intermediate byproducts observed in the degradation of MB are 2-amino-5-(N-methyl formamide) benzene sulfonic acid, 2-amino-5-(methyl amino)-hydroxybenzene sulfonic acid, benzenesulfonic acid, and phenol (Fig. 12e).

1 $$ - {\text{ln}}\left( {{\text{C}}/{\text{C}}_{{\text{o}}} } \right) = - {\text{kt}} $$

where C_0_ and C is the initial and apparent concentration of the MB and k is the kinetic rate constant. The values of k were obtained from the slope and the intercept of the linear plot. Figure 12d shows a linear relationship between ln(C/C_0_) and the irradiation time for MB degradation. The rate constants for MB photodegradation with Ni-SDZ under visible light are − 0.01634 min^−1^. The values of k indicated that the activity of photocatalyst Ni-SDZ is better than that of reported data^58,91,98–100^.

## Conclusion

In conclusion, the Ni(II) complex of sulfadiazine has been successfully synthesized. The magnetic measurements (EPR and magnetic susceptibility) data reveal the complex coordination is distorted octahedral. By crystal data results, the sulfadiazine ligand is bidentate and the geometry of the Ni-SDZ molecule is suggested to be octahedral, involving two nitrogen atoms of the sulfonamide group and two nitrogen atoms from the 3-methyl pyridine ligand. The experimental results were complemented with DFT calculations showing a good agreement between calculated and experimental geometrical parameters. The NBO analysis reveals that the intra-molecular interaction orbital overlaps providing the maximum stability to the molecule. Hirshfeld surface analysis reveals intermolecular O···H and H···H contacts are the most significant interactions in the crystal. The DNA interaction studies as performed using relative viscosity measurement and UV–visible absorption titration suggest intercalation of the complex in between the stacks of DNA base pairs. The molecular docking study suggests the minor groove as the first site of interaction of complex for DNA followed by intercalation. The cytotoxic and cell viability analysis of the complex suggests the potent cytotoxic nature of the complex. The molecular docking study complemented the DNA binding studies of the complex. The percentage of degradation of methylene blue dye was found to be 70.19% within 70 min of visible light radiation.

### Supplementary Information

Supplementary Information.

## Data Availability

We would like to confirm that all relevant data and materials supporting the findings of our journal paper are available upon request. For data inquiries, please contact Bhavesh Socha at bhaveshkumarsocha@gmail.com.
