# Supplementary material for: Visible light-driven photocatalysts, quantum chemical calculations, ADMET-SAR parameters, and DNA binding studies of nickel complex of sulfadiazine
Source: Sci Rep. 2023 Sep 15;13:15275. doi: 10.1038/s41598-023-42668-z (PMC10504334; doi:10.1038/s41598-023-42668-z)
Supplement: Supplementary file 1 — Supplementary Information. [file 41598_2023_42668_MOESM1_ESM.docx]

**Supplementary Informations**

**Figure S 1** Chemical diagram of sulfadiazine (SDZ) ligand

**Figure S 2** FT-IR spectra of SDZ and its complex Ni-**SDZ**

**
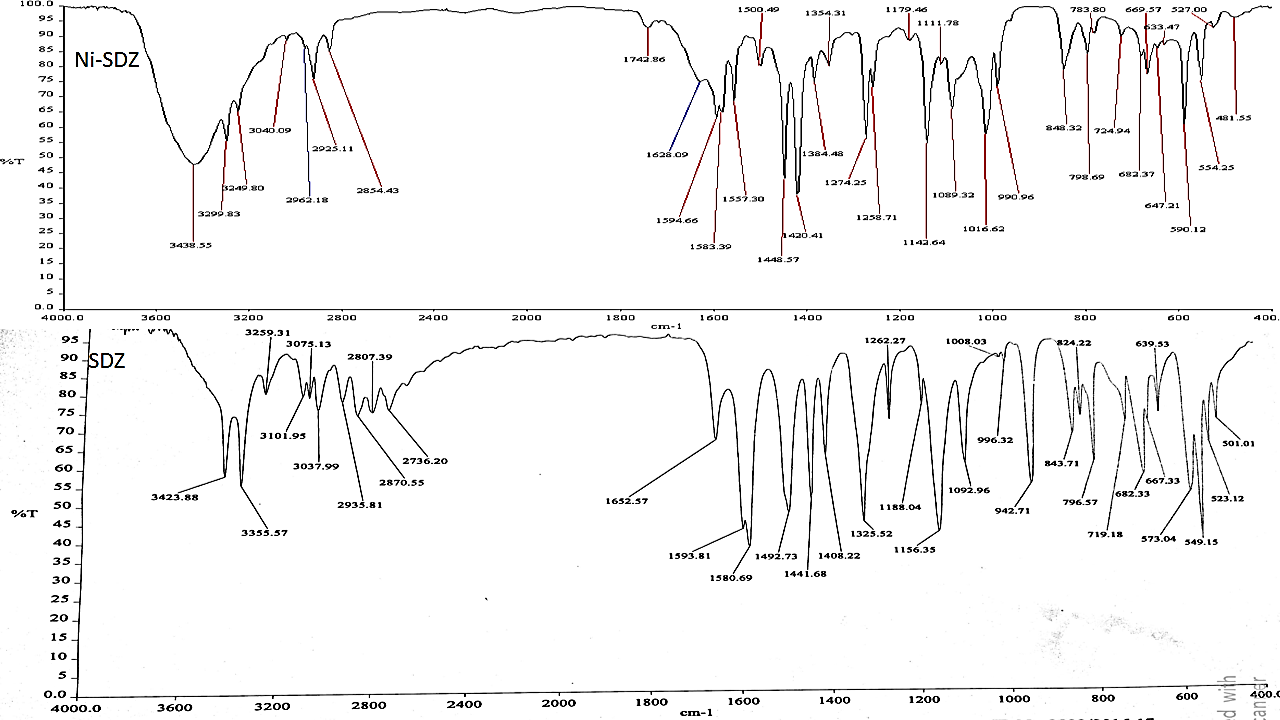
**

**Figure S 3** Magnetic susceptibility of nickel complex of SDZ at R.T. (293K)


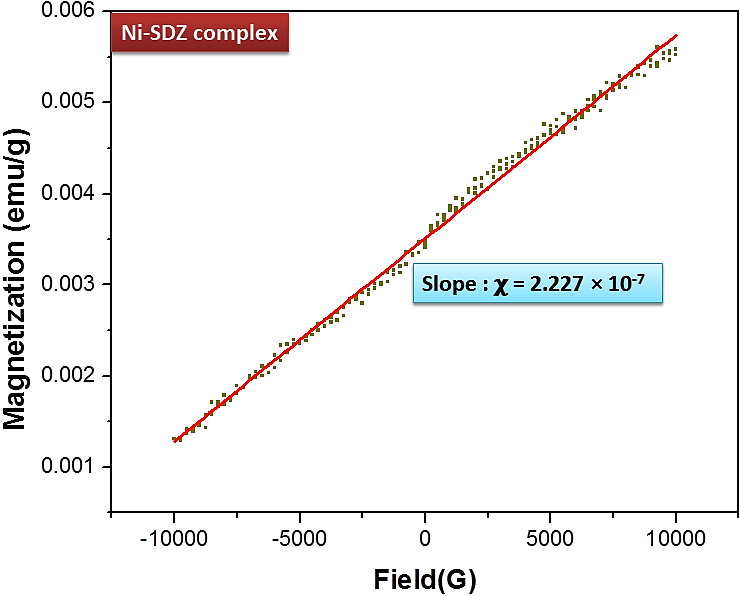


**Figure S 4** Graphical representation of correlation between experimental versus theoretical bond lengths and bond angles of Ni-SDZ molecule


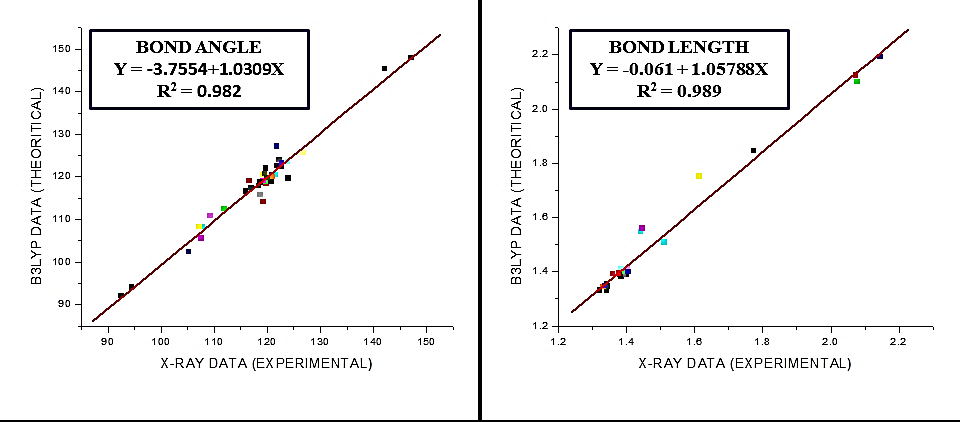


**Table S 1** Mullikan population charges of each atom in Ni-SDZ

| **Atom** | **Charge** | **Atom** | **Charge** |
| --- | --- | --- | --- |
| Ni1 | 0.41883 | C19 | -0.35513 |
| S2 | 1.65838 | C20 | -0.00532 |
| O3 | -0.74922 | C22 | -0.14545 |
| O4 | -0.80557 | C24 | 0.2426 |
| N5 | -0.47617 | C25 | -0.15011 |
| N6 | -0.39928 | C27 | -0.01545 |
| N7 | -0.80577 | C29 | 0.07584 |
| N8 | -0.59597 | C31 | -0.09028 |
| N11 | -0.44978 | C33 | -0.08189 |
| C12 | 0.10252 | C35 | 0.09708 |
| C14 | -0.13554 | C36 | 0.04969 |
| C16 | 0.08734 | C38 | -0.39093 |
| C18 | 0.65447 |  |  |

**Figure S 5** Relative viscosity of DNA with increasing amount of complex
